# Supplementary material for: Assessing the relationship between male alcohol consumption and intimate partner violence against women in Sub-Saharan Africa: A dual-dataset secondary analysis of recent DHS surveys
Source: PLoS One. 2026 Apr 21;21(4):e0331422. doi: 10.1371/journal.pone.0331422 (PMC13098983; doi:10.1371/journal.pone.0331422)
Supplement: Attachment — Submitted filename: Point-by-point response.docx [file pone.0331422.s002.docx]

**Point-by-point response to the reviewer**

Dear Dr. Mikler,
Thank you for your insightful and valuable feedback, which has greatly strengthened our article. We have revised the manuscript accordingly and would appreciate your review of the updated document.

1. Data analysis

The sampling procedure requires special procedure called weighted complex sampling. This is important for the estimation of appropriate standard errors because of nested sampling. Why were the authors did not conduct complex sampling weighted analysis?

**Response**: Thank you for your important question. This approach has been addressed in the analysis, and the results are presented in the results section. We have also clarified this further in the manuscript. Specifically, we included the following statement:

*"The data were weighted to ensure representativeness and to provide more accurate statistical estimates. We accounted for the complex survey design features of the DHS—namely sampling weights, clustering, and stratification—in all our analyses to ensure that the effect estimates were unbiased and representative at both national and sub-regional levels. In the pooled analyses, we applied de-normalized sampling weights based on each country’s population size and additionally adjusted for country and survey year fixed effects to control for unobserved country-specific factors and temporal trends”.*

2. The study outcomes sexual/physical/emotional and pooled IPV are all binary(proportions)

For regression analysis, I would expect them to conduct logistic regression, not linear models which are used if the outcome variable is a continuous variable. Please clarify.

**Response**: This isa great point. However, the traditional logistic regression model from a cross-sectional study may overestimate the relative risk when the outcome is common and in case of convergence issues modified Poisson regression model performs better in estimating the prevalence ratio. the description is included in the methodology section with references.

“We used the modified Poisson regression models with robust error variance and reported the results as an adjusted prevalence ratio with a 95% confidence interval. This model modified the Posson regression model since the odds ratio estimated using the traditional logistic regression from a cross-sectional study may overestimate the relative risk when the outcome is common [1, 2] and in case of convergence issues this model performs better in estimating the prevalence ratio [3, 4]”.

3. Need details on the process on how the variables were entered. Were they entered all at once or used stepwise regression option?

**Response**: we used the modified poisson regression model and hence we entered all variables simultaneously.

- Variables

4. Although the authors provided the reference, they should provide details on how pooled IPV and pooled alcohol consumption were computed. Did they add all 5 countries and take the average?

**Response**: thank you for your valuable comment. We have modified this section in the methodology part.

The IPV is defined as women who had experienced at least one event of physical, emotional or sexual violence since the age of 15 years [5-7].

| Table 1: The tool used to measure IPV in the Demographic and Health Surveys. | |
| --- | --- |
| Question/item | IPV type |
| Push you, shake you, or throw something at you? | physical IPV |
| Slap you? |  |
| Twist your arm or pull your hair |  |
| Punch you with his/her first or with something that could hurt you? |  |
| Kick you, drag you, or beat you up? |  |
| Try to choke you or burn you on purpose? |  |
| Threaten or attack you with a knife, gun or any other weapon? |  |
| Physically force you to have sexual intercourse with him even when you did not want to? | Sexual IPV |
| Physically force you to perform any other sexual acts you did not want to? |  |
| Force you with threats or in any other way to perform sexual acts you did not want to? |  |
| Say or do something to humiliate you in front of others? | Emotional IPV |
| Threaten to hurt or harm you or someone close to you? |  |
| Insult you or make you feel bad about yourself? |  |

Multiple regression model:

5. How did the authors choose which variables to include in the model? Is it based on the bivariate associations presented in Table 2? Please include degrees of freedom and p-value for each crosstabulation. This is important for the reader to see these values. Or use asterisks to indicate the level of significance. *p<.05; ** p=< .01; *** p<.001

**Response**: This is a good point. However, we used (answered in no. 3) modified poission regression model and we didn’t use the crude and adjusted binary logistic regression models.

6. Use variables that have a significant association with IPV at the bi-variate level.

There are some independent variables in the model that might be highly associated (multicollinearity). For example, women afraid of husband and partners controlling behavior; working status and wealth index; Country and districts , etc.

Women might be afraid because of husband’s controlling behavior. It has a direct effect.

Check for multicollinearity.

**Response**: We checked the Variance Inflation Factor (VIF) to the variables and it ranged from 1.12 to 3.65 which reveals there was no multicolinearity effect (157-58).

Results/
7. Second paragraph… Begin with “Table 2 presents bivariate associations between study variables and the outcome.”

**Response**: thanks again, it is corrected (162).
8. Discussion-
The question on IPV asks about violence experienced since the age of 15 years. It is possible that they might not have experienced violence with the current husband. So, it is likely that they might be reporting incidents occurred with their prior partners/boyfriends or their experiences with other family members (incest). We cannot establish a direct link to IPV and the prevalence of alcohol consumption of the current partner.
We cannot establish a direct link unless the partner was under the influence when the IPV event occurred. So, the authors need to be careful in claiming direct causation between alcohol and committing violence. Please indicate this fact under limitations.

**Response**: we appreciate it, the published DHS reports showed 3-10% of adults got divorce and established new relationships in the region. Hence, we included a limitation statement at the end of the discussion stating that, “A limitation of this study is that participants who experienced intimate partner violence (IPV) in a previous relationship may report these incidents when describing their current relationship, potentially confounding the findings” (265-68).

9. Please do not repeat the numbers that are already presented in the tables.
   Need to add study limitations section and how they might influence the study findings.
   Please include your study implications as you see fit for Sub Sahara African region.

**Response**: we revised it accordingly. Also, we amend implications to the policymakers, programmers and researchers (274-79).

10. Tables:

Include sample size (N) for all the tables and subgroups.
Table 1: delete % symbol next to each number. Put a note at the bottom of the table saying Note: The prevalence is presented in percentage along with 95% confidence intervals in the parenthesis.
Table 2 : please include degrees of freedom and p-value for each crosstabulation. Or use asterisks to indicate the significance level and add a note at the bottom of the table.
This is important for the reader to see the bivariate association between the study variables and the outcome types of IPV.
Table 3: CI values are missing for education under emotional IPV.
Table 3 title needs to be changed to reflect what is presented.
“Results from multivariable regression predicting odds of IPV” do not use the term “correlation” correlation is a statistical term measuring association between two continuous variables.Note at the bottom:
Note: Adjusted odds ratios and 95% Confidence Intervals are presented in parenthesis.
Add a note at the bottom of the table for the asterisks to indicate the significance level.
*p<.05; ** p=< .01; *** p<.001

**Response**: thanks for the critical review, we revised all the tables accordingly.

References

1.	Barros, A.J. and V.N. Hirakata, *Alternatives for logistic regression in cross-sectional studies: an empirical comparison of models that directly estimate the prevalence ratio.* BMC medical research methodology, 2003. **3**: p. 1-13.

2.	Tamhane, A.R., et al., *Prevalence odds ratio versus prevalence ratio: choice comes with consequences.* Statistics in medicine, 2016. **35**(30): p. 5730-5735.

3.	Yelland, L.N., A.B. Salter, and P. Ryan, *Performance of the modified Poisson regression approach for estimating relative risks from clustered prospective data.* American journal of epidemiology, 2011. **174**(8): p. 984-992.

4.	Zou, G., *A modified poisson regression approach to prospective studies with binary data.* American journal of epidemiology, 2004. **159**(7): p. 702-706.

5.	Chernet, A.G. and K.T. Cherie, *Prevalence of intimate partner violence against women and associated factors in Ethiopia.* BMC women's health, 2020. **20**(1): p. 1-7.

6.	Straus, M.A. and E.M. Douglas, *A short form of the Revised Conflict Tactics Scales, and typologies for severity and mutuality.* Violence and victims, 2004. **19**(5): p. 507-520.

7.	Temmerman, M., *Research priorities to address violence against women and girls.* The Lancet, 2015. **385**(9978): p. e38-e40.
